# Probiotic Properties and Antioxidant Activity In Vitro of Lactic Acid Bacteria

**DOI:** 10.3390/microorganisms11051264

**Published:** 2023-05-11

**Authors:** Despina Vougiouklaki, Theofania Tsironi, Andreas G. Tsantes, Efstathia Tsakali, Jan F. M. Van Impe, Dimitra Houhoula

**Affiliations:** 1 Department of Food Science and Technology, Faculty of Food Sciences, University of West Attica, 12461 Egaleo, Greece; 2 Department of Food Science and Human Nutrition, Agricultural University of Athens, 11855 Athens, Greece; 3 Laboratory of Haematology and Blood Bank Unit, School of Medicine, ‘Attiko’ Hospital, National and Kapodistrian University of Athens, 12462 Athens, Greece; 4 Department of Chemical Engineering, BioTeC+—Chemical and Biochemical Process Technology and Control, KU Leuven, 9000 Gent, Belgium

**Keywords:** lactic acid bacteria, LAB, antioxidant activity, gastrointestinal tract, probiotics, bacteriocin

## Abstract

The properties of probiotics such as lactic acid bacteria (LAB) have been widely studied over the last decades. In the present study, four different LAB species, namely *Lactobacillus gasseri* ATCC 33323, *Lacticaseibacillus rhamnosus* GG ATCC 53103, *Levilactobacillus brevis* ATCC 8287, and *Lactiplantibacillus plantarum* ATCC 14917, were investigated in order to determine their ability to survive in the human gut. They were evaluated based on their tolerance to acids, resistance to simulated gastrointestinal conditions, antibiotic resistance, and the identification of genes encoding bacteriocin production. All four tested strains demonstrated high resistance to simulated gastric juice after 3 h, and the viable counts revealed declines in cell concentrations of less than 1 log cycle. *L. plantarum* showed the highest level of survival in the human gut, with counts of 7.09 log CFU/mL. For the species *L. rhamnosus* and *L. brevis*, the values were 6.97 and 6.52, respectively. *L. gasseri*, after 12 h, showed a 3.96 log cycle drop in viable counts. None of the evaluated strains inhibited resistance to ampicillin, gentamicin, kanamycin, streptomycin, erythromycin, clindamycin, tetracycline, or chloramphenicol. With regard to bacteriocin genes, the Pediocin PA gene was identified in *Lactiplantibacillus plantarum* ATCC 14917, *Lacticaseibacillus rhamnosus* GG ATCC 53103, and *Lactobacillus gasseri* ATCC 33323. The PlnEF gene was detected in *Lactiplantibacillus plantarum* ATCC 14917 and *Lacticaseibacillus rhamnosus* GG ATCC 53103. The Brevicin 174A and PlnA genes were not detected in any bacteria. Moreover, the potential antioxidant activity of LAB’s metabolites was evaluated. At the same time, the possible antioxidant activity of metabolites of LAB was first tested using the free radical DDPH^•^ (a, a-Diphenyl-β-Picrylhydrazyl) and then evaluated with regard to their radical scavenging activity and inhibition against peroxyl radical induced DNA scission. All strains showed antioxidant activity; however, the best antioxidant activity was achieved by *L. brevis* (94.47%) and *L. gasseri* (91.29%) at 210 min. This study provides a comprehensive approach to the action of these LAB and their use in the food industry.

## 1. Introduction

Probiotics have been a relevant scientific issue over the last few decades, and several aspects of probiotics have been studied. According to the FAO/WHO (Food and Agricultural Organization of United Nations/World Health Organization), probiotics are defined as “live microorganisms which when consumed in adequate amounts as part of food confer a health benefit on the host” [1]. In this way, they have the ability to regulate the microbial balance in the gastrointestinal tract [1,2,3]. They have several functional mechanisms, including affections on strengthening the epithelial barrier, inhibition of pathogens through antagonism, binding and interaction with the host, and the derivation of antimicrobials [4].

A wide range of food products—mainly dairy and/or fermented foods—are good providers of probiotics for humans [1,5,6,7]. Lactic acid bacteria (LAB) are mostly used in fermentation processes [8] as they provide lactic acid through carbohydrate conversion and also contribute to the development of the unique sensory characteristics of fermented food products. In addition, they assist in the safety of food products through the production of antimicrobial agents with action against pathogens such as *Listeria monocytogenes, Salmonella enterica, Escherichia coli, Klebsiella pneumoniae*, and *Staphylococcus aureus* [8,9]. The most common probiotics that are either naturally encountered or used in these food products belong to the genera of *Lactobacillus* and *Bifidobacterium*, which have been classified as safe (GRAS) [10]. They are mainly saccharolytic, Gram-positive, rod shaped, and reside in the large bowel [1,3,11]. They have an aerotolerant anaerobic nature [6], and thus they can be found in non-aerobic environments, but they also can support aerobic conditions [2,5,12].

On the other hand, some LAB strains are vital in the digestive tract, producing antimicrobial metabolites such as bacteriocins and preventing pathogenic and infectious microorganisms from growing. Thus, a set of criteria are suggested to prove probiotic activity, including the ability to provide desirable metabolites (such as bacteriocins) with tolerance to acid and bile salt, as well as adherence ability in the intestinal tract and a lack of resistance to antibiotics [10,13,14]. Several LAB, especially the *Lactobacillus* genera, produce bacteriocins, which are antimicrobial peptides that are different from antibiotics in that they act on closely related microorganisms [15,16,17,18]. They can be basically categorized as Class I Lantibiotics (such as nicin and lactocin), Class II Non Lantiotics (such as pediocin PA1 and leucocin A), and Class III Bacteriotoxins (such as pantaricin A and enterotoxin A) [15,18].

LAB bacteriocin genes can be accurately identified based on genome sequencing. The bacteriocin encoding genes are found in operon clusters placed on the chromosome (such as PlantaricinST31), plasmids (such as Plantaricin423), or transposons (such as Nisin A). Several studies have identified bacteriocins from different strains, including Plantaricin from *L. plantarum* (C11, WCFS1, NC8, J23 and J51), Pediocin PA-1/AcH from *L. plantarum* WHE 92, Sakacin674 from *L. sake* 674, SakacinP from *L. sake* LTH673, and Bavaricin A from *L. bavaricus* MI401 [19].

Development of antibiotic resistance, over the past years, has been an issue of great significance, and a lot of research is being carried out on this topic [6,8,19]. The European Food Safety Authority (EFSA) suggests that any bacteria added on purpose into the food chain should not have acquired resistance determinants to antimicrobials of clinical importance [7,20]. There is always the case of intrinsic or natural resistance to antimicrobials from all strains of certain species; acquired resistance refers to the resistance of a certain strains due to genes obtained through exogenous DNA or the mutation of indigenous genes [10,21].

Antioxidant activity of several agents has been extensively studied, and the scientific interest has turned to natural antioxidants due to their therapeutic and health promoting abilities [22,23]. The levels of reactive oxygen species (ROS) are crucial for the immune response against invading microbes, but, on the other hand, the risk of oxidative stress is present in cases of over-ROS production, leading to protein and lipid oxidation and DNA damage [24,25,26,27]. LAB have been extensively studied for their antioxidant activity using different experimental approaches. Their antioxidant effect has been demonstrated and includes free radical-scavenging capacities, lipid peroxidation–inhibition capacities, and metal-chelating abilities, along with a variety of antioxidant enzyme activities [24,28]. Members of the *Bifidobacterium* and *Lactobacillus* genera have been reported to decrease the levels of 2,2-diphenyl-1-picrylhydrazyl (DPPH) and 2,2′-azino-bis (3-ethylbenzothiazoline-6-sulfonic acid) (ABTS) free radicals [24,29,30,31,32]. Thus, an appropriate approach to exploring the antioxidant activity of a certain strain can be performed by evaluating DPPH and ABTS radical scavenging activity and nitric oxide inhibition [24].

As there is a lack of data, the contribution of the present study is the characterization of the probiotic properties of certain LAB, namely *Lactobacillus gasseri* ATCC 33323, *Lacticaseibacillus rhamnosus* GG ATCC 53103, *Levilactobacillus brevis* ATCC 8287, and *Lactiplantibacillus plantarum* ATCC 14917. The evaluation is based on their tolerance to acids, resistance to simulated gastrointestinal conditions, antibiotic resistance, identification of genes encoding bacteriocin production, and their antioxidant activity as per their radical scavenging activity and inhibition against peroxyl radical induced DNA scission. 

## 2. Materials and Methods

### 2.1. Bacterial Species and Culture Conditions 

For the cultivation of the studied species *Lactobacillus gasseri* ATCC 33323, *Lacticaseibacillus rhamnosus* GG ATCC 53103, *Levilactobacillus brevis* ATCC 8287, and *Lactiplantibacillus plantarum* ATCC 14917 (LAB), MRS broth (De Man Rogosa Sharp) was used. The incubation took place at 37 °C for 48 h under anaerobic conditions, and stock cultures in MRS broth containing 20% (*v/v*) sterile glycerol were kept at −80 °C [33,34].

### 2.2. Extraction Procedures 

The extraction procedure was based on the steps descripted in previous works [33,34], and it was as follows: The cultivation and incubation took place as described above, and it was followed by centrifugation of the broth at 13,000× *g* for 7 min. In order to achieve cell-free supernatants (CFSs), the supernatants were filtered with a medium of 0.22 μm pore size while the bacteria free non-inoculated MRS broth medium was used as a negative control.

### 2.3. Liquid Liquid Extraction (LLE)

In 50 mL conical tubes containing 10 mL ethyl acetate, 1 g NaCl, 4 g Na_2_SO_4_, and 10 mL CFSs were added. They were centrifuged at 4000× *g* for 10 min. A volume of 5 mL of the organic phase was collected by adding 100 μL of dimethyl sulfoxide (DMSO) and letting it dry in a rotary evaporator followed by reconstitution of the dry phase with 90% water and 10% methanol before the sample was filtered (0.22 μm) [33,34].

### 2.4. Assay Antioxidant Activity of LAB Species In Vitro 

#### Scavenging of a, a-Diphenyl-β-Picrylhydrazyl (DDPH) Free Radical

The DDPH^•^ is a violet-colored, stable free radical, which is reduced to 2,2-diphenyl-1-picrylhydrazine (pale yellow) by reacting with an antioxidant [34,35,36]. The DDPH^•^ solution of 6 × 10^−5^ M was prepared in methanol. A volume of 1.0 mL of CFSs was added to 2.5 mL of ethanolic DPPH radical solution (A_sample_). The sample was incubated at room temperature (RT) in the dark for 30 min after mixing vigorously. Absorbance of the supernatant was measured at 517 nm in triplicate, and the CFSs free DDPH radical was also measured (A_control_). Total antioxidant activity was expressed in μg/mL gallic acid, and the results were expressed as the amount of antioxidant needed to cause a 50% reduction in DDPH absorption (IC50). In order to compute the radical scavenging activity, the following equation was used:

Radical scavenging activity (%) = [(A_control_ − A_sample_)/A_control_] × 100%

where A_sample_ = absorbance of sample; A_control_ = absorbance of control.

Standard solutions of DL-p-Hydroxyphenyllactic acid (OH-PLA), 1,2-dihydroxybenzene, benzoic acid, salicylic acid, vanillic acid, ferulic acid, and 4-hydro-cinnamic acid were used in order to evaluate the antioxidant activity.

### 2.5. Inhibition against Peroxyl Radical Induced DNA Scission

The effectiveness of LAB CFSs against DNA cleavage was determined according to a modification of the method described by Brown et al. [37]. DNA strand breaks were induced by 2,2′-Azobis (2-amidinopropane) dihydrochloride (AAPH) and examined by agarose electrophoresis. A buffer containing a mixture of Tris base, acetic acid, and EDTA (TAE buffer, pH 8.5) at a concentration of 25 μg/mL was used to suspend DNA. A volume of 4 μL of supercoiled pBR322 DNA, 4 μL of 30 mM AAPH, and 2 μL of sample diluted in 10 mM PBS (phosphate-buffered saline) were mixed and incubated at 37 °C for 30 min, and a blank (no sample) and a control (no AAPH or sample) were also prepared. In each mixture. A volume of 1 μL of loading dye (gel loading dye purple (6×), no SDS, (New England BioLabs, Ipswich, MA, USA)) was added before the samples were loaded onto a 0.8% agarose gel with 15 μL ethidium bromide. Electrophoresis was performed at 100 V for approximately 70 min, and the bands were visualized under UV light. The intensity of the DNA bands was measured using the UV illumination MiniBIS Pro device (DNR Bio-Imaging Systems Ltd., Modi’in-Maccabim-Re’ut, Israel). Antioxidant activity was expressed as the percentage of DNA that remained intact in the sheared DNA normalized against the control [37].

### 2.6. Evaluation of Probiotic Properties

#### 2.6.1. Tolerance to Acids 

A volume of 10 mL PBS (pH 2.5) was mixed with LAB species (10^8^ CFU/mL), and they were incubated at 37 °C for 1, 2, and 3 h. Viable counts were then determined on MRS agar plates. The biomass (CFU/mL) of each culture obtained in the assays, made in triplicate, was enumerated on MRS agar incubated anaerobically at 37 °C for 3 days [38]. The formula below was used to obtain the survival rate (%).

Survival rate (%) = Biomass after treatment by acid (C1)/Biomass at initial time (C0) × 100

#### 2.6.2. Resistance to Simulated Gastrointestinal Conditions 

Simulated gastric and intestinal fluids were prepared according to published reports [24,38]. In total, 1 g pepsin (pepsin, from porcine gastric mucosa, Sigma-Aldrich, St. Louis, MO, USA), 1.5 g gastric mucin (mucin from porcine stomach Type II, Sigma-Aldrich), 8.7 g NaCl, and 5 g bile salts (bile extract porcine, Sigma-Aldrich) were dissolved in 1 L water. After 3 h, 1 mL of the suspension was inoculated into 9 mL of simulated intestinal fluid (pH 8.0 and incubated at 37 °C). The viability (log cfu/mL) of *Lacticaseibacillus rhamnosus* GG ATCC 53103, *Lactiplantibacillus plantarum* ATCC14917, *Lactobacillus gasseri* ATCC 14917, and *Levilactobacillus brevis* ATCC 8287 was tested after 3, 6, 9, and 12 h of incubation at pH 8 (HCl 2M) in the presence of 0.5% (*w/v*) bile salts derived from dried pig bile. The samples were incubated anaerobically at 37 °C and retrieved for enumeration at their respective end points.

#### 2.6.3. Antibiotic Resistance 

To attain a density of 10^8^ CFU/mL for inoculating to the solid media and incubating at 37 °C under anaerobic conditions for 24 h, cells were cultured in MRS broth at 37 °C for 24 h. After that, 0.5 McFarland of an inoculum was made. A continuous antibiotic gradient was created in the agar medium as a result of the homogenous inoculation of an agar plate, application of the strip, and prompt release of the ETEST Gradient strip technology (Etest, bioMérieux, Craponne, France) from the carrier. An ellipse of growth inhibition was produced after incubation, and the minimum inhibitory concentration (MIC) was calculated at the point where the ellipse crossed the scale on the upper side of the strip. For each of the following substances: ampicillin, gentamicin, kanamycin, streptomycin, erythromycin, clindamycin, tetracycline, and chloramphenicol, the MIC of the antimicrobials expressed as g/mL was determined [39]. 

### 2.7. Genomic DNA Extraction 

LAB were cultivated anaerobically for 48 h in MRS broth at 37 °C, and they were then centrifuged at 14,000× *g* for 15 min. Following the supplier’s recommended procedure, DNA was immediately extracted from the cell pellet using an automatic extractor and the Nucleic Acid Extraction Kit (ZYBIO Corporation, Chongqing, China). Using an Epoch spectrophotometer from Biotek Winooski, Winooski, VT, USA, the quality and quantity of isolated DNA were assessed spectrophotometrically by calculating the OD260/OD280 ratio [34].

### 2.8. Identification of Genes Encoding Bacteriocin Production 

PCR was used to identify the genes encoding bacteriocin production, according to Azizi et al. [19] after certain changes. The following conditions were used for plnA, plnEF, and pediocin PA-1 gene amplification: 95 °C for 5 min, then 30 cycles of 94 °C for 30 s of denaturation, 1 min of annealing at various temperatures listed in Table 1, 1 min of extension, and 10 min of final extension at 72 °C. Brevicin 174A gene PCR was performed as follows: 1 cycle of 5 min at 96 °C, 15 s at 58 °C, and 30 s at 72 °C was followed by 29 cycles of 1 min at 96 °C, 15 s at 58 °C, and 30 s at 72 °C, and a final extension of 7 min at 72 °C. Table 1 lists the specific primers applied in this investigation. PCR products were examined on a 2.0% (*w/v*) agarose gel with ethidium bromide staining (0.5 g/mL) from Sigma, Kanagawa, Japan. As a benchmark for molecular weight, a 100 bp ladder (Invitrogen, Paisley, UK) was utilized. The MiniBIS Pro device (DNR Bio- Imaging Systems Ltd., Neve Yamin, Israel) was used to document gels under UV illumination after running them for about 1 h at 120 V [19].

### 2.9. Statistical Analysis

The antioxidant activity of the tested probiotics was analyzed using analysis of variance (ANOVA) with a 95% level of significance (STATISTICA^®^ 7.0, StatSoft Inc., Tulsa, OK, USA). Duncan’s multiple range test “*p* = 0.05” was used to calculate the significant differences. For the statistical fit of exponential models (two phases decay) to the experimental data of DPPH scavenging rate, non-linear regression was used (XLSTAT 2023.1.1). 

## 3. Results and Discussion

In order to provide their beneficial effect, probiotic species should be viable following consumption and remain resistant to the hostile conditions of the gastrointestinal system. There are several studies reporting certain criteria for selection of probiotic strains based on their resistance to bile salts and low pH environments. Moreover, criteria related to the safety for human use and antibiotic resistance of the strains are particularly important. 

Based on the results of our previous research [33,34], we chose four species of lactic acid bacteria with antimicrobial action against pathogenic food microorganisms. However, although this antimicrobial action is a required condition for their probiotic potential, it does not establish them as probiotic strains. For this reason, several properties of these strains were evaluated to further elaborate their use as probiotic strains.

### 3.1. Resistance to Simulated Gastric and Intestinal Fluids

The stomach’s pH ranges from 1.5 to 4.5, and ingestion takes about 3 h. For probiotic microorganisms to survive passage through the stomach, acid tolerance is a crucial trait. In this study, an initial screening method was used under acidic conditions (pH 2.5 for 3 h). All four tested strains demonstrated high resistance to simulated gastric juice, as shown in Table 2. After 3 h, all strains were still viable, and the viable counts revealed declines in cell concentrations of less than 1 log cycle. These four strains were therefore chosen for additional research. A potential probiotic must be able to survive passage through the digestive system and sufficiently populate the colon.

As shown in Table 3, the three species (*Lactiplantibacillus plantarum* ATCC 14917, *Lacticaseibacillus rhamnosus* GG ATCC 53103, and *Levilactobacillus brevis* ATCC 8287) showed good survival capacity after exposure to simulated intestinal juice. Therefore, these three species were selected for further studies. Among them, *Lactiplantibacillus plantarum* ATCC 14917 showed the highest level of survival with counts of 7.09 log CFU/mL. For the species *Lacticaseibacillus rhamnosus* GG ATCC 53103 and *Levilactobacillus brevis* ATCC 8287, the values were 6.97 and 6.52, respectively. *Lactobacillus gasseri* ATCC 33323, after 12 h, showed a 3.96 log cycle drop in viable counts. The cell populations remained over 4 log CFU/mL. In contrast to gastric juice, the *Lactobacillus gasseri* was sensitive to intestine juice. 

### 3.2. Gradient Concentration Strip (Etest) Method 

The probiotic species must be suitable for consumption by humans. Genes that cause antibiotic resistance are typically found on plasmids. Pathogenic bacteria with a high level of antibiotic resistance may develop if such plasmids are conjugated with other bacteria. In this study, the susceptibility of four LAB strains to eight antibiotics was evaluated. The outcomes are shown in Table 4. The microbiological cut-off values established by the EFSA Panel on Additives and Products or Substances used in Animal Feed [21] were used to determine the susceptibility of species. The investigation was carried out three times. Based on the results, these probiotic species as safe for consumption.

### 3.3. Antioxidant Activity In Vitro of LAB

**Figure 1:**
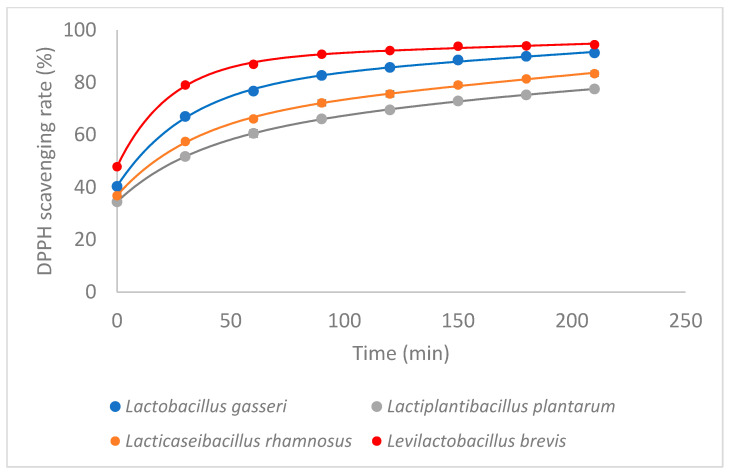
Antioxidant activity of LABs (after 5 days incubation). (Experimental data and statistical fit of Exponential equations—Two phases decay = pr1 × exp(−pr2 × X1) + pr3 × exp(−pr4 × X1) + pr5).

The nitrogen bridge contains one atom of the DPPH free radical, which is a stable radical with an unpaired valence electron. The popular DPPH antioxidant assay is based on the scavenging of DPPH radicals. An imbalance between oxidant and antioxidant actions causes oxidative stress. In our study, the DPPH radical scavenging technique was used to assess the in vitro antioxidant activity of CFS from LAB. At various times (0 min, 30 min, 60 min, 90 min, 120 min, 150 min, 180 min, 210 min), the absorbance of various CFSs from four LAB concentrations was measured. All LAB has DDPH radical scavenging activity. Comparing all the lactic acid bacteria, the best antioxidant activity was achieved by *L. brevis* (94.47%) and *L. gasseri* (91.29%) at 210 min. The scavenging rate of DPPH of *L. rhamnosus* and *L. plantarum* was 83.41% and 77.53% at 210 min. In previous research [33,34], we had identified and quantified the metabolites produced in the strains *Lactobacillus gasseri* ATCC 33323, *Lacticaseibacillus rhamnosus* GG ATCC 53103, *Levilactobacillus brevis* ATCC8287, and *Lactiplantibacillus plantarum* ATCC 14917. Initially, we studied the antioxidant activity of CFS of each lactic acid bacteria after 5 days incubation and the results showed that all species have antioxidant activity (Figure 1).

**Figure 2:**
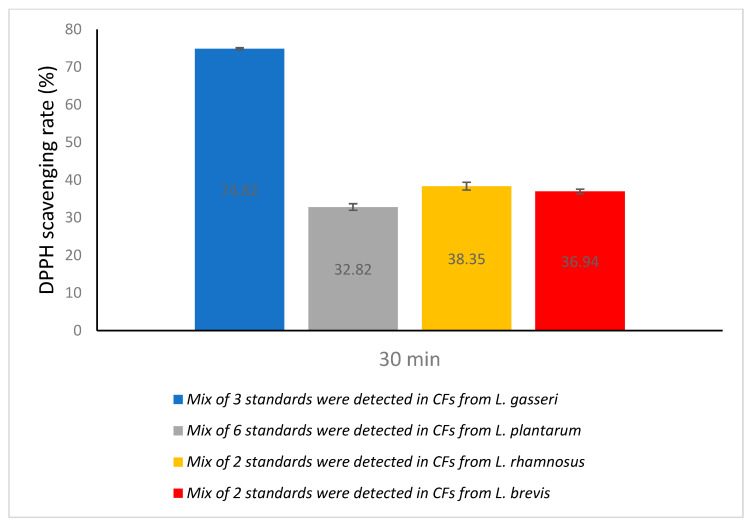
Antioxidant activity of standards we detected in LABs.

We then tested the metabolites identified in each lactic acid bacteria separately and recorded the quantities after 5 days of incubation. The metabolites detected in *Lactobacillus gasseri* ATCC 33323 were DL-p-Hydroxyphenyllactic acid (OH-PLA) (6.0 ppm), 1,2-dihydroxybenzene (3.73 ppm), and benzoic acid (2.31 ppm). OH-PLA (150.3 ppm), salicylic acid (1.0 ppm), vanillic acid (2.2 ppm), ferulic acid (4.8 ppm), benzoic acid (3.2 ppm), and 4-hydro-cinnamic acid (1.0 ppm) were identified in *Lactiplantibacillus plantarum* ATCC 14917. OH-PLA (123.2 ppm) and ferulic acid (3.85 ppm) were found in *Lacticaseibacillus rhamnosus* GG ATCC 53103, as well as OH-PLA (80.2 ppm) and vanillic acid (1.5 ppm) in *Levilactobacillus brevis* ATCC 8287. At the same time, the antioxidant activity of the standards that were detected and quantified in CFS from each lactic acid bacteria was studied. The scavenging rate of DPPH of the mix of 3 standards ((OH-PLA), 1,2-dihydroxybenzene, benzoic acid) was 74.82% (Figure 2). On the other hand, the 6 standard mixtures, OH-PLA, salicylic acid, vanillic acid, ferulic acid, benzoic acid, and 4-hydro-cinnamic acid, showed a scavenging rate of 32.82%. The 2 standard mixtures OH-PLA and ferulic acid showed a scavenging rate of 38.35%. The 2 standard mixtures OH-PLA and vanillic acid showed a scavenging rate of 36.94%. As shown from the results, there are other antioxidant metabolites in CFS of the four LAB that increase the scavenging rate. 

### 3.4. Inhibition against Peroxyl Radical Induced DNA Scission

**Figure 3:**
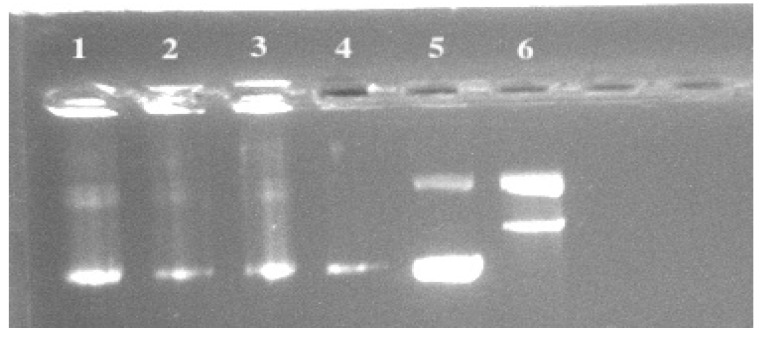
Peroxyl radical induced DNA scission gels in the presence of CFSs of LAB. Line 1: CFS of *Levilactobacillus brevis* ATCC 8287, Line 2: CFS of *Lacticaseibacillus rhamnosus* GG ATCC 53103, Line 3: CFS of *Lactobacillus gasseri* ATCC 33323, Line 4: CFS of *Lactiplantibacillus plantarum* ATCC 14917, Line 5: control (DNA only), Line 6: Blank (DNA and AAPH only).

The supercoiled plasmid DNA strand inhibition assay gauges how well samples guard against peroxyl radicals cutting DNA strands. Figure 3 shows the outcomes for DNA cleavage inhibition. The inhibition of DNA cleavage largely followed the predicted pattern. The highest activity was seen in the CFS of *Lactiplantibacillus plantarum* ATCC 14917, which had a 97.38% inhibition rate. Next in line were the CFS of *Levilactobacillus brevis* ATCC 8287, *Lacticaseibacillus rhamnosus* GG ATCC 53103, and *Lactobacillus gasseri* ATCC 33323, with 94.15%, 89.21%, and 88.29% inhibition rates. The control (only DNA) revealed about 15% cleaved DNA, whereas the blank (only DNA and AAPH) displayed no inhibition. The control (DNA only) revealed about 15% nicked DNA, whereas the blank (DNA and AAPH only) displayed no inhibition.

### 3.5. Detection of Bacteriocin Structural Genes 

**Figure 4:**
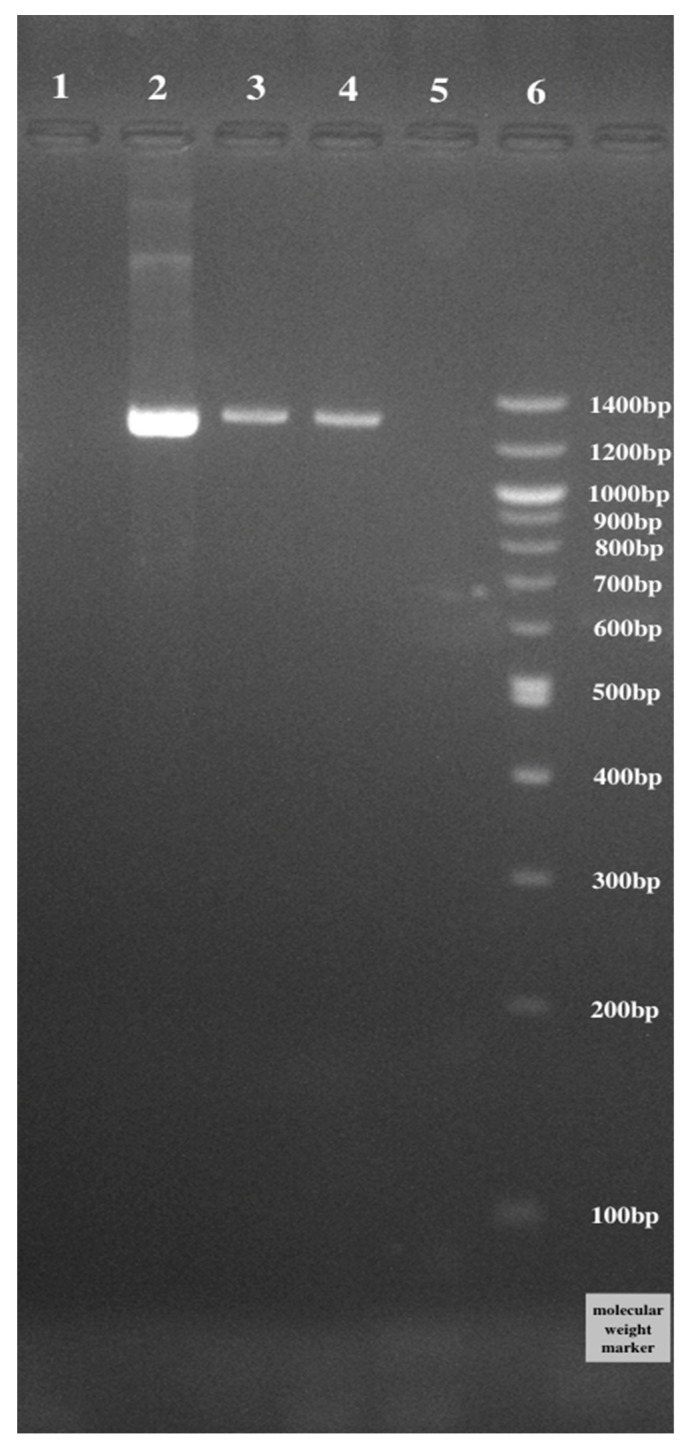
Detection of Pediocin PA (1220 bp) sequences in LAB. Line 1: *Levilactobacillus brevis* ATCC 8287; Line 2: *Lactiplantibacillus plantarum* ATCC 14917; Line 3: *Lacticaseibacillus rhamnosus* GG ATCC 53103; Line 4: *Lactobacillus gasseri* ATCC 33323; Line 5: negative; Line 6: ladder.

**Figure 5:**
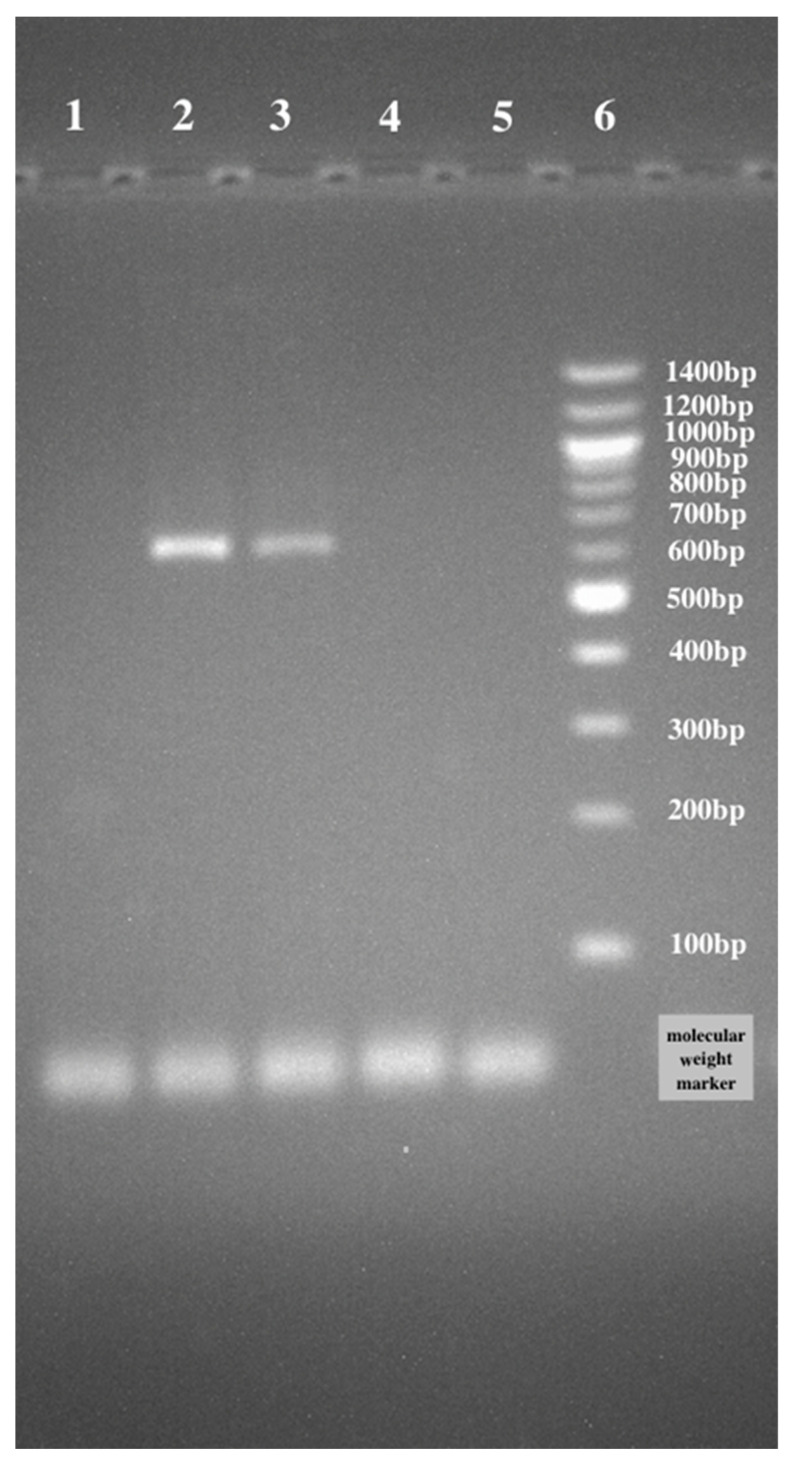
Detection of plnEF (616 bp) sequences in LAB. Line 1: *Levilactobacillus brevis* ATCC 8287; Line 2: *Lactiplantibacillus plantarum* ATCC 14917; Line 3: *Lacticaseibacillus rhamnosus* GG ATCC 53103; Line 4: *Lactobacillus gasseri* ATCC 33323; Line 5: negative; Line 6: ladder.

In order to detect the presence of genes encoding bacteriocin, PCR reactions were performed using four sets of specific primers. Products of 616 and 1220 bp were detected using specific primers of Pediocin PA-1 and plnEF, respectively. However, no DNA fragment was amplified using the specific primer of plnA and brevicin174A (Table 5). According to Figure 4, the Pediocin PA gene was present in *Lactiplantibacillus plantarum* ATCC 14917, *Lacticaseibacillus rhamnosus* GG ATCC 53103, and *Lactobacillus gasseri* ATCC 33323. In addition, the presence of the plnEF gene in *Lactiplantibacillus plantarum* ATCC 14917 and *Lacticaseibacillus rhamnosus* GG ATCC 53103 (Figure 5) was detected.

Azizi et al. (2017) [19] investigated the presence of the above genes in 11 different species of lactic acid bacteria (*L. plantarum* M16, *L. plantarum* M17, *L. plantarum* M18, *L. plantarum* M19, *L. brevis* M1, *L. brevis* M2, *L. brevis* M6, *L. brevis* M7, *L. brevis* M8, *L. brevis* M10, and *L. brevis* M12). The results of the study showed that only *L. plantarum* M16, *L. plantarum* M17, *L. plantarum* M18, and *L. plantarum* M19 contained the genes PlnA and PlnEF. However, no DNA fragment found in any bacteria was amplified using the Pediocin PA-1 specific primer. In addition, the Bre174A gene was identified in *L. brevis* M1, *L. brevis* M7, *L. brevis* M8, *L. brevis* M10, and *L. brevis* M12. 

## 4. Conclusions

In summary, our data suggests that lactic acid bacteria are excellent sources of natural antioxidants, possessing antimicrobial activity against foodborne pathogens. Additionally, these species may be used as starter cultures in fermented foods in the future. In our study, a series of in vitro analyses were used to evaluate the probiotic properties of four LAB species. All species exhibited optimum probiotic properties with the exception of *Lactobacillus gasseri* ATCC 33323, which presented the lowest microbial population dynamic after 12 h of incubation in the intestinal juice. Moreover, none of the evaluated strains inhibited resistance to ampicillin, gentamicin, kanamycin, streptomycin, erythromycin, clindamycin, tetracycline, or chloramphenicol. The results of the present study could assist in the prevention of the bacterial resistance spread. Further studies are needed to evaluate certain characteristics of these probiotic strains, such as growth and survival in food matrixes. In addition, further investigations are needed to determine whether specific lactic acid bacteria strains possess in vivo activities before their use as additives in food products can be recommended.

## Figures and Tables

**Table 1:** Primers used throughout this study and their amplification details.

Name	Sequence (5′ → 3′)	Size Amplicon	Annealing Temperature	References
Brevicin 174A-F	GTCTTAAATGCTAGGCTTGTCA	766	58	[19]
Brevicin 174A-R	CTGGCAAGACAAACGGTTAG			
PlnA-F	TAGAAATAATTCCTCCGTACTTC	573	57	
PlnA-R	ATTAGCGATGTAGTGTCATCCA			
plnEF-F	TATGAATTGAAAGGGTCCGT	616	56	
plnEF-R	GTTCCAAATAACATCATACAAGG			
Pediocin PA-1-F	AAAGATACTGCGTTGATAGG	1220	50	
Pediocin PA-1-R	GAGAAGCCATGCTGAAAG			

**Table 2:** Survival rate of the LAB strains in simulated gastric juice.

Species	Initial Log (CFU/mL)	Gastric Juice		
		1 h	2 h	3 h
*Lactobacillus gasseri* ATCC 33323	7.97	7.96 (99.87%)	7.89 (99.0%)	7.83 (98.24%)
*Lactiplantibacillus plantarum* ATCC 14917	7.94	7.92 (99.75%)	7.86 (98.99%)	7.84 (98.74%)
*Lacticaseibacillus rhamnosus* GG ATCC 53103	8.00	7.99 (99.86%)	7.99 (99.86%)	7.91 (98.86%)
*Levilactobacillus brevis* ATCC 8287	8.02	8.00 (99.75%)	7.91 (98.63%)	7.88 (98.25%)

**Table 3:** Survival rate of the LAB species in simulated intestinal juice.

Species	Initial Log (CFU/mL)	Intestinal Juice			
		3 h	6 h	9 h	12 h
*Lactobacillus gasseri* ATCC 33323	7.83	7.02 (89.65%)	6.15 (78.55%)	5.82 (74.33%)	3.87 (49.43%)
*Lactiplantibacillus plantarum* ATCC 14917	7.84	7.56 (96.42%)	7.39 (94.26%)	7.24 (92.35%)	7.09 (90.43%)
*Lacticaseibacillus rhamnosus* GG ATCC 53103	7.91	7.67 (96.97%)	7.42 (93.81%)	7.13 (90.14%)	6.97 (88.12%)
*Levilactobacillus brevis* ATCC 8287	7.88	7.53 (95.56%)	7.13 (90.48%)	6.87 (87.18%)	6.52 (82.74%)

**Table 4:** Distribution of MICs of tested antibiotics among phenotypically resistant LAB strains (n = 4).

Species	MIC (μg/mL)							
	GM	K	TE	CH	A	E	CL	S
Microbiological cut-off values (μg/mL) proposed by EFSA for obligate heterofermentative *Lactobacillus*								
	16	32	8	4	2	1	1	64
*Lactobacillus gasseri*	14	28	6	3	1	1	1	64
*Levilactobacillus brevis*	1	16	3	2	0.125	0.50	0.32	32
Microbiological cut-off values (μg/mL) proposed by EFSA for *Lactobacillus plantarum/pentosus*								
	16	64	32	8	2	1	2	n.r
*Lactiplantibacillus plantarum*	4	12	4	3	0.25	0.75	0.25	-
Microbiological cut-off values (μg/mL) proposed by EFSA for *Lactobacillus rhamnosus*								
	16	64	8	4	4	1	4	32
*Lacticaseibacillus rhamnosus*	8	42	0.75	0.38	2	0.75	2	24

**Table 5:** PCR amplification of bacteriocin genes from lactic acid bacteria.

Bacteriocinogenic Isolates	Bacteriocin Gene			
	PlnA	PlnEF	Pediocin PA-1	Bre174A
*Levilactobacillus brevis* ATCC 8287	-	-	-	-
*Lactiplantibacillus plantarum* ATCC 14917	-	+	+	-
*Lacticaseibacillus rhamnosus* GG ATCC 53103	-	+	+	-
*Lactobacillus gasseri* ATCC 33323	-	-	+	-

(+) gene present (-) no presence of gene.
